# Integrin α1 Has a Long Helix, Extending from the Transmembrane Region to the Cytoplasmic Tail in Detergent Micelles

**DOI:** 10.1371/journal.pone.0062954

**Published:** 2013-04-30

**Authors:** Chaohua Lai, Xiaoxi Liu, Changlin Tian, Fangming Wu

**Affiliations:** 1 Hefei National Laboratory for Physical Science at Microscale and School of Life Sciences, University of Science and Technology of China, Hefei, Anhui, China; 2 High Magnetic Field Laboratory, Chinese Academy of Sciences, Hefei, Anhui, China; University of Birmingham, United Kingdom

## Abstract

Integrin proteins are very important adhesion receptors that mediate cell-cell and cell-extracellular matrix interactions. They play essential roles in cell signaling and the regulation of cellular shape, motility, and the cell cycle. Here, the transmembrane and cytoplasmic (TMC) domains of integrin α1 and β1 were over-expressed and purified in detergent micelles. The structure and backbone relaxations of α1-TMC in LDAO micelles were determined and analyzed using solution NMR. A long helix, extending from the transmembrane region to the cytoplasmic tail, was observed in α1-TMC. Structural comparisons of α1-TMC with reported αIIb-TMC domains indicated different conformations in the transmembrane regions and cytoplasmic tails. An NMR titration experiment indicated weak interactions between α1-TMC and β1-TMC through several α1-TMC residues located at its N-terminal juxta-transmembrane region and C-terminal extended helix region.

## Introduction

Integrins are cell adhesion receptors that mediate cell-cell and cell-extracellular matrix interactions, regulating cell growth and function. These receptors transmit bidirectional signals across the plasma membrane and contribute to the regulation of development, immune responses, inflammation, hemostasis, and to the development of many human diseases, including infection, autoimmunity, and cancers [1], [2], [3], [4]. They are hetero-dimeric, type I transmembrane proteins consisting of α and β subunits. Each subunit contains a relatively large extracellular domain, a single transmembrane domain (TM), and a short cytoplasmic tail (CT) [4]. In mammals, 18 α subunits and 8 β subunits can form 24 different hetero-dimers that are expressed in particular tissues and bind to particular ligands. There are two forms of integrin signaling, which are known as outside-in and inside-out signaling.

A subgroup of collagen integrin receptors, α1/β1, α2/β1, α10/β1, and α11/β1, mediate cell adhesion to extracellular matrix [5]. They have a similar collagen-binding αI domain, but have different ligand binding mechanisms and collagen subtype specificities [6], [7], [8]. Integrin α1/β1 has been found to participate in the regulation of fibrosis [9], cancer-related angiogenesis [10], chronic inflammation [11], the development of myopia [12], and in the homing and differentiation of prostate cancer stem cells [2].

Structural characterizations of the extracellular domains of integrins have long been studied [13], [14], [15], [16]. However, few studies on the transmembrane and cytoplasmic (TMC) domains of integrins have been reported. In recent years, the TM and TMC domains of integrin αIIb and β3 were studied, alone or in complex, in organic solvents, detergent micelles, bicelles, or lipids using NMR (nuclear magnetic resonance) methods [3], [17], [18], [19], [20], [21], [22]. Also, interaction interfaces between αIIb/β3 TM helices were studied using cysteine scanning and disulfide bond formation methods [23]. Multiple hydrophobic and electrostatic contacts within the membrane proximal helices of αIIb and β3 were revealed [3]. However, very few reports about the structures of integrin α1-TMC and β1-TMC are available.

Here, integrin α1-TMC (G1135-K1179) and β1-TMC (V717-K798) were over-expressed using a bacterial system and purified in LDAO (lauryl-dimenthylamine-n-oxide) detergent micelles. The solution structure of α1-TMC in detergent micelles was determined using NMR. The structure determined showed a long helix, extending from the transmembrane region to the cytoplasmic tail of integrin α1-TMC, which differed from the previously reported structure of integrin αIIb-TMC. Backbone ^15^N relaxation data for α1-TMC in LDAO micelles also confirmed the extended helix. A chemical shift perturbation study of α1-TMC with the addition of integrin β1-TMC illustrated intensity attenuation in aqueous/membrane interfacial residues of integrin α1-TMC, indicating weak interactions between integrin α1-TMC and β1-TMC at these residues.

## Materials and Methods

### Cloning and Over-expression of Human Integrin α1/β1 TMC

Synthetic oligonucleotides encoding integrin α1-TMC (G1135-K1179) and β1-TMC (V717-K798) were amplified and subcloned into expression vector pET21b (Novagen) with a C-terminal 6×His-tag. The recombinant protein was expressed using BL21(DE3) Gold in M9 medium at 25°C for 15 h. To achieve over-expression of isotope-labeled integrin α1-TMC, 1 g/L ^15^N-NH_4_Cl and 3 g/L ^13^C-D-glucose (Cambridge Isotope Laboratory) were used as the sole nitrogen and carbon sources, respectively.

### Purification of Integrin α1/β1 TMC in Detergent Micelles

Cells were harvested by centrifugation and suspended in lysis buffer (70 mM Tris-HCl, 300 mM NaCl, pH 8.0), then lysed by sonication (Sonics and Materials), incubated with lysozyme (1.0 mg/mL), DNase (0.02 mg/mL), RNase (0.02 mg/mL), and magnesium acetate (5 mM) at 4°C for 2 h. After centrifugation, the supernatant was discarded and the pellet was washed twice in lysis buffer. The pellet was suspended in binding buffer (20 mM Tris, 100 mM NaCl, pH 8.0) in the presence of 1% SDS (sodium dodecyl sulfate) (w/v) and incubated at room temperature for 30 min, followed by centrifugation (40,000 g, 20 min, 18°C). The pellet was discarded and the supernatant was diluted using binding buffer until the concentration of SDS reached 0.2% (w/v). The protein was purified using a Ni^2+^-NTA (Qiagen) gravity-flow column, which was washed using washing buffer (20 mM Tris, 100 mM NaCl, pH 8.0, 0.2% (w/v) SDS). Then, binding buffer with 0.2% (w/v) LDAO (Anatrace) was used to exchange detergents and achieve on-column protein refolding. Proteins were eluted using elution buffer (20 mM Tris, 100 mM NaCl, 0.5% LDAO, 250 mM imidazole, pH 8.0). Amicon Ultra-15 centrifugal filter units (Millipore) were used to remove imidazole and concentrate the protein. The final NMR sample contained 1.0 mM integrin α1-TMC, 50 mM NaH_2_PO_4_-Na_2_HPO_4_ (pH 6.5), 10% (v/v) D_2_O, 2 mM dithiothreitol (DTT), and 250 mM LDAO. The concentration of the protein was determined by OD_280_, and the purity was analyzed using SDS-PAGE (polyacrylamide gel electrophoresis).

### Solution NMR Spectroscopy of Human Integrin α1-TMC

A set of multi-dimensional NMR experiments of ^15^N- or^ 13^C/^15^N-labeled integrin α1-TMC were conducted at 30°C, using a 600 MHz Bruker spectrometer equipped with a TXI cryo-probe. NMR spectra, including HSQC (hetero-nuclear single quantum correlation spectroscopy), HNCO, HNCA, HNCACB, CBCA(CO)NH, CC(CO)NH, HBHA(CO)NH, and HCC(CO)NH, were collected to obtain chemical shift assignments of backbone and side chain atoms. ^15^N-edited NOESY-HSQC spectra (mixing time 100 ms) were collected to confirm the chemical shift assignments and to generate distance restraints for structure calculations. All NMR spectra were processed using NMRPipe [24] and analyzed using NMRView [25].

### Residual Dipolar Coupling (RDC) Experiment

For backbone amide RDC measurements, a 6.5% polyacrylamide gel was prepared. Liquid gels (300 µL) will polymerize overnight at room temperature in 6-mm Teflon casting tubes. The polymerized gels were incubated for 2 h in 5 mL RDC buffer (50 mM NaH_2_PO_4_-Na_2_HPO_4_, pH 6.5) and then in 5 mL RDC buffer, supplemented with 0.5% LDAO and 10% D_2_O. Then, the gel was incubated with 2 mL ^15^N-labeled integrin α1-TMC sample in the same detergent/buffer solution at room temperature for 2 days. The protein-soaked gel was then stretched into a 5-mm NMR tube using a device similar to that developed by the Bax group [8].

One-bond ^1^H-^15^N RDCs [26] were measured by acquiring a pair of spectra to yield semi-TROSY (TROSY in the ^1^H dimension and anti-TROSY in the ^15^N dimension) and semi-TROSY (TROSY in the ^15^N dimension and anti-TROSY in the ^1^H dimension) resonances using ^15^N-labeled integrin α1-TMC in stretched gels. The couplings were obtained from the ^15^N resonance frequency differences of the two semi-TROSY contour peak components.

### Structure Calculations

Backbone dihedral angle restraints were obtained from the backbone chemical shifts using TALOS+ [27]. Extensive side chain NMR resonance assignments were not possible, such that the ^1^H-^1^H NOEs (nuclear Overhauser effect) used to derive distance restraints for structural calculation were limited primarily to short-range backbone HN-HN distances. Backbone dihedral angle, NOE, and one-bond ^1^H-^15^N RDC restraints were used to calculate the structure of integrin α1-TMC with Xplor-NIH [28]. The final ten structures with the lowest energy were verified using PROCHECK-NMR [29]. Chemical shifts have been deposited in BioMagResBank (accession 17424). The structural coordinates have been deposited in PDB (accession 2L8S).

### Backbone ^15^N Relaxation Measurements

A ^15^N-labeled integrin α1-TMC sample was used for ^1^H-^15^N relaxation data collection on a 500 MHz Bruker spectrometer. Backbone ^15^N longitudinal relaxation T_1_ values were determined from a series of ^1^H-^15^N correlation spectra with 11.2, 61.6, 142, 243, 364, 525, 757, and 1150 ms relaxation evolution delays. Backbone ^15^N transverse relaxation T_2_ values were obtained from the spectra with 0, 17.6, 35.2, 52.8, 70.4, 105.6, and 140.8 ms delays. Steady-state ^1^H-^15^N NOE values were determined from peak ratios observed between two spectra collected with or without a 3 s power presaturation in the proton channel.

### NMR Titration of Integrin α1-TMC with β1-TMC in LDAO

Non-labeled integrin β1-TMC was used to titrate the ^15^N-labeled α1-TMC. During the titration, the molar ratios of α1/β1 were 1∶0, 1∶0.5, and 1∶1. The spectra were collected on a Varian 500 MHz NMR spectrometer at 30°C.

## Results and Discussion

### Primary Sequence Analysis of the TMC Domains of Different Integrins

The transmembrane regions of the 18 α integrins are well-conserved, as shown in Figure S1A. Integrin α1 has the shortest C-terminus among all α subunits and has a specific PLKKKMEK polybasic sequence [30]. A conserved GFFKR motif in α subunits is considered to be an interaction site between integrin α1 and β1, similar to the combination of hydrophobic and electrostatic interactions between αIIb and β3 [3]. This conserved GFFKR motif is known to play an important role in the regulation of integrin function, while deletion of the specific PLKKKMEK sequence has been reported to affect α1/β1-dependent signal transduction [30]. A tentative topology map of integrin α1-TMC, including the transmembrane helix, is shown in Figure S1B, with the conserved GFFKR motif highlighted.

### Solution NMR Backbone Resonance Assignment of Integrin α1-TMC

**Figure 1:**
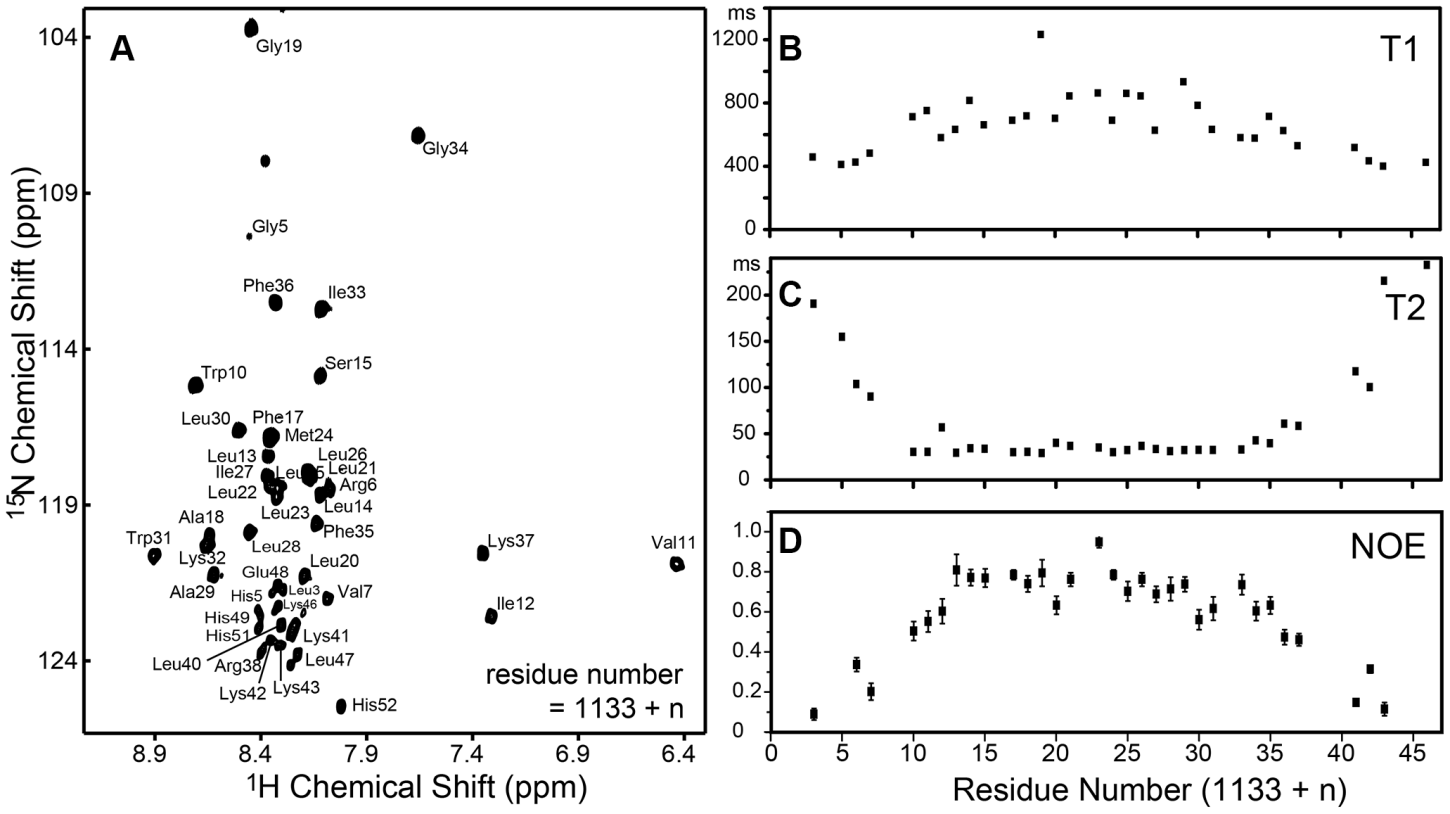
Resonance assignment and Backbone ^15^N relaxation analysis of integrin α1-TMC in LDAO micelles. (A) Resonance assignment of integrin α1-TMC in LDAO micelles. Site-specific analysis of backbone amide ^15^N longitudinal relaxation T1 (B), transverse relaxation T2 (C) and steady-state ^1^H-^15^N NOE (D) of integrin α1-TMC in LDAO micelles.

A high-quality HSQC spectrum for α1-TMC was obtained in LDAO micelles, which was the basis for further resonance assignments and structural determination of the protein in LDAO. With collection of a full set of triple resonance and three-dimensional solution NMR spectra, sequential resonance assignments were achieved for backbone nuclei (^13^C_α_, ^13^C_β_, ^13^CO, amide ^15^N/^1^H) of integrin α1-TMC in LDAO micelles. The HSQC spectrum with each resonance assigned to residues of integrin α1-TMC is shown in Figure 1A. In total, 43 sets of backbone carbon resonances (including ^13^CO, ^13^C_α_ and ^13^C_β_) and 38 backbone amide (^1^H, ^15^N) resonances were assigned. There were still four residues (G1135, L1142, M1177, E1178) that could not be assigned, probably due to peak overlap of the narrowly dispersed HSQC spectrum of the sample in detergent micelles, or microsecond-millisecond motion of these residues.

The secondary structure of integrin α1-TMC in LDAO micelles was analyzed using TALOS+ [27] from assigned chemical shift values of ^13^CO, ^13^C_α_, ^13^C_β_, amide ^1^H, and ^15^N (Fig. S2). Site-specific secondary structure prediction indicated that, in total, 24 residues (L1142-K1165) were shown in an α-helix secondary structure, corresponding to the transmembrane helix of integrin α1-TMC.

### Structural Calculation and Description

**Figure 2:**
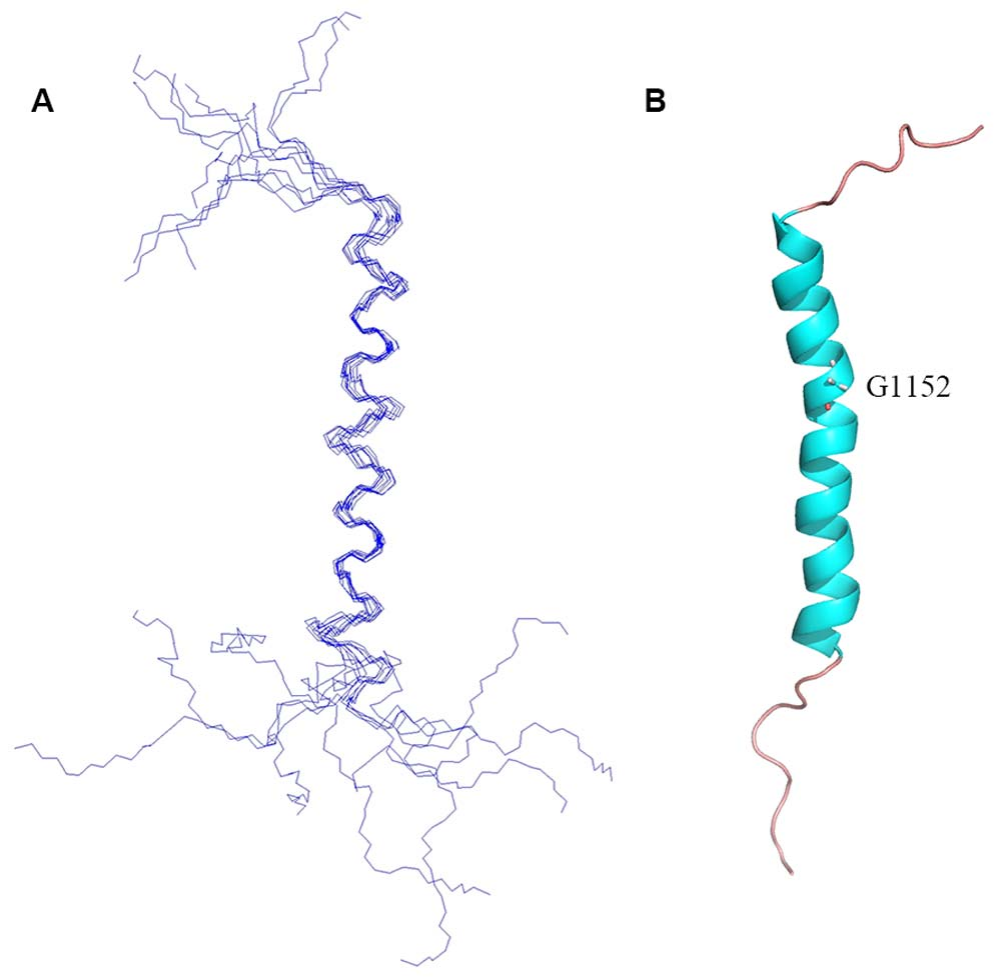
Solution NMR structure of human integrin α1-TMC in LDAO micelles. (A) The backbone superposition of the final ten structures with the lowest energies. (B) Cartoon representation of the structure of integrin α1-TMC. G1152 indicates the position of the transmembrane helix kink.

The solution structure of integrin α1-TMC was determined using Xplor-NIH [28], based on 212 NOE, 60 dihedral angle, and 32 backbone ^1^H-^15^N RDC restraints. Ten lowest energy structures were selected out of 100 calculated structures. Structural computation statistics regarding the quality and precision of integrin α1-TMC are summarized in Table 1. The backbone superimposition of the final ten conformers is presented in Figure 2A. In this structure, a kink was observed in the transmembrane helix at the position of G1152 (Fig. 2B). A stretch of helix with 28 residues was observed to extend from the transmembrane helix to the conserved GFF motif. This conformation of the integrin α1-TMC domain in LDAO micelles was also consistent with the backbone ^15^N relaxation data (Fig. 1B–D). The longitudinal relaxation T_2_ values of residues W1143-F1168 were similar (about 30 ms; Fig. 1C) and their steady-state NOE values were above 0.5 (Fig. 1D), indicating that these residues (W1143-F1168) form a stable secondary structural region, flanked by two flexible terminals.

**Table 1:** Structural Statistics for the Final 10 Conformers of Human Integrin α1-TMC.

*Structural restraints*	
NOE distance restraints	212
Intraresidue	58
Sequential	93
Medium-range (2≤|i-j|≤4)	61
Long-range (|i-j|≥5)	0
TALOS dihedral angle constraints	60
Backbone ^1^H-^15^N RDC	32
Total	304
***Statistics for 10 structures***	
RMSD from experimental restraints	
Distance	0.0530±0.0092
Dihedral	2.5069±0.2551
RMSD from idealized covalent geometry	
Bonds (Å)	0.0021±0.0003
Angles (deg)	0.5211±0.0169
Impropers (deg)	0.5731±0.0294
***Coordinate precision***	
RMSD to the mean (Å)	Backbone/Heavy atoms
Residues in secondary structure elements (9–36)	0.730/1.406
Residues of TM Helix (9–31)	0.525/1.088
***Ramachandran plot statistics (%)***	
Most favored regions	81.9
Additional allowed regions	11.4
Generously allowed regions	5.7
Disallowed regions	1.1

### Structural Comparison of Integrin α1-TMC with other Reported Integrin TMC Domains

Using solution NMR or computation modeling methods, several structures of integrin transmembrane helix (TM) and/or C-terminal tails (CT) have been determined over the past 10 years. Most of the reported integrin TM or CT structures are integrin αIIb/β3, which play important roles in primary platelet adhesion [3], [19], [20], [21], [22], [31], [32]. Previously, several TM or CT domain structures of integrin αIIb/β3 have been studied in different amphipathic environments (such as detergent micelles, phospholipid bicelles) or organic solvents. Some minor structural differences were observed for the same integrin segments in the different environments.

**Figure 3:**
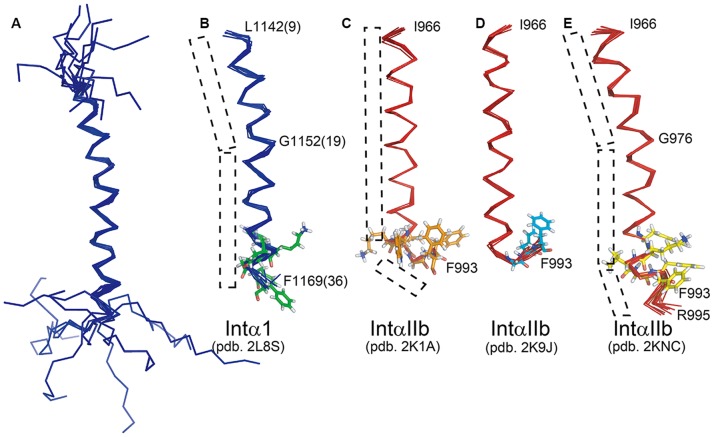
Structural comparison of integrin α1 and αIIb. Structure of α1-TMC (1135–1179) and backbone structure comparisons of α1-TM(1142–1169) with αIIb-TM structures indicate different bent regions in the transmembrane helix. The PDB number for each structure is listed below. (A) Structure ensemble of integrin α1-TMC in LDAO micelles; (B) Structure ensemble of integrin α1-TM in LDAO; (C) αIIb-TM (966–993) in bicelles [22]; (D) αIIb-TM from IntαIIb/β3 complex in bicelles [20]; (E) αIIb-TM (966–993) from αIIb/β3 complex in organic/aqueous solvents, 50% CD_3_CN/50% H_2_O [19].

Here, the structure of integrin α1-TMC in LDAO micelles was compared with several representative structures of αIIb, such as the αIIb TM segment in bicelles (PDB: 2K1A) [22], the αIIb/β3 TM complex in bicelles (PDB: 2K9J) [20], and the αIIb/β3 TMC complex in CD_3_CN/H_2_O (PDB: 2KNC) [19]. In Figure 3, the three αIIb structures were shown, alongside our α1 TMC structure. Clearly, a kink was observed in both integrin α1-TMC at Gly1152 (Fig. 3B) and integrin αIIb-TMC of the αIIb/β3 TMC complex in CD_3_CN/H_2_O (Fig. 3E, G976), while no such kink was seen in the structure of integrin αIIb-TMC in bicelles (Fig. 3C, 3D).

Notably, in the cytoplasmic region of integrin α1 and αIIb, the GFF (1167-GFF-1169 in α1 and 991-GFF-993 in αIIb) has been reported to form a helix in detergent and organic solvent (Fig. 3B, 3E) [19], or a GFF reverse turn with its two Phe residues immersed back into the hydrophobic region of bicelles (Fig. 3C, 3D) [20], [22]. Moreover, the GFF helical conformation [33] and a GFF reverse turn [31], [34] can be readily obtained through different computation modeling. In particular, a CS-Rosetta prediction by Yang *et al*. showed that the GFF reverse turn was the majority conformation, while the GFF helical conformation was seen in a small proportion [19]. In addition to the two different conformations of αIIb/β3 in different conditions [19], [20], integrin α/β heterodimer formation efficiencies were also affected by different membrane-mimicking environments [35]. In light of these results and the complex physiological function of integrins, it is possible that integrin α1-TMC could be in multiple conformations (helix or reverse turn). Transitions between different conformations could be induced by environmental changes and/or specific physiological processes (e.g., activation/inactivation or monomer/dimer formation).

On the other hand, it was previously reported that the conserved GFFKR motifs in different integrins have different correlations with their functions. For example, the αIIb F992A or F993A mutation can activate αIIb/β3 [36] while the FF/AA mutation in this motif had little effect in the activation of αV/β3 [37]. Probably, the conformations of the GFFKR motif in these two integrins are different. Previously, it was reported that the αIIb/β3 association was sensitive to the integrity of the αIIb(R995)–β3(D723) salt bridge [20], [36], the KR residues in GFFKR motif having undefined structure might provide some flexibility for the salt bridge formation between integrin α1 and integrin β1.

Here, an extending helical conformation of integrin α1-TMC was determined using solution NMR in detergent micelles, indicating a majority helical conformation of integrin α1-TMC in its monomeric form in micelles. Whether a conformation with a GFF reverse turn can be observed in the α1/β1 complex or in lipid bilayers need to be examined in future studies.

### Interactions between Integrin α1-TMC and β1-TMC in LDAO Micelles

**Figure 4:**
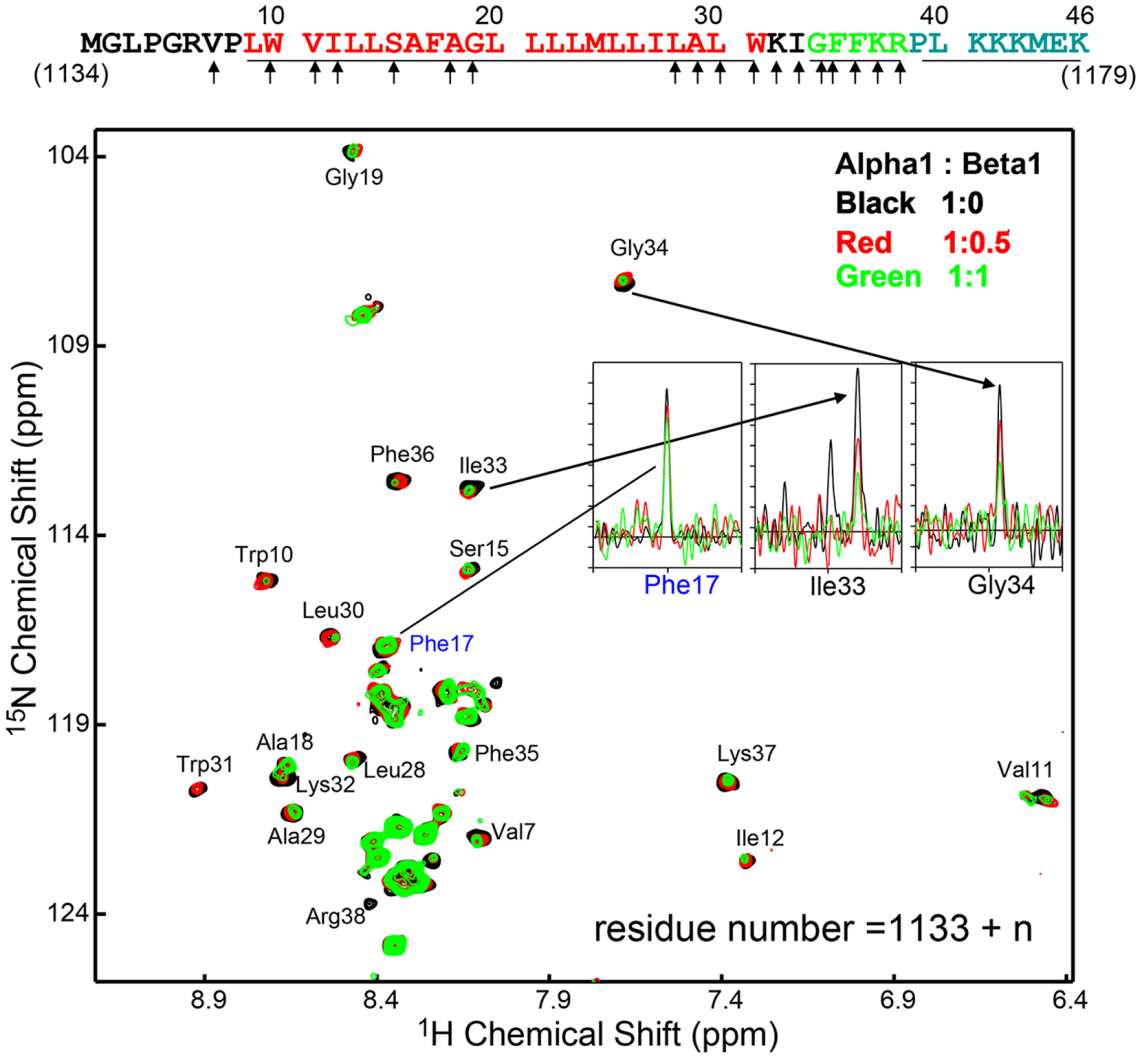
Chemical shift perturbation analysis of integrin α1-TMC upon the addition of integrin β1-TMC. HSQC titration of^ 15^N-labeled integrin α1-TMC with addition of unlabeled integrin β1-TMC. Some peaks of integrin α1-TMC are missing at elevated integrin β1-TMC concentrations (marked with residue names). Residues with missing peaks are probably due to intermediate time-scale interactions between α1-TMC/β1-TMC, consistent with predicted interaction points of α1 and β1. A peak intensity with little change is exemplified with F17 and missing peaks are shown for I33 and G34.

Interactions between the TMC domain of integrin α1 and β1 are known to be important for cell adhesion, probably due to integrin clustering. The C-terminal tail of integrin α1 plays an essential role in both physiological and pathological angiogenesis [38]. Deletion of the entire cytoplasmic tail of integrin α1 or mutations in several amino acids distal to the highly conserved GFFKR motif have been reported to have a similar phenotype to parental α1-null cells, resulting in malfunctions in angiogenesis and endothelial cell proliferation [38]. The highly conserved GFFKR motif in the α1 tail has been proposed to form a salt bridge with conserved residues in the β1 tail. However, the detailed interaction between TMC domains of α1 and β1 is not yet understood. Due to the complex enthalpy solvent effects of detergent mixing or exchange between two membrane protein samples, isothermal titration calorimetry assay is not suitable for analyzing interactions between α1-TMC/β1-TMC in detergent micelles. Thus, NMR titration experiment was employed to study the interaction between α1-TMC and β1-TMC. First of all, we acquired the HSQC spectrum of β1-TMC to make sure it’s a well folded sample (Fig. S3). Then, a series of ^1^H-^15^N HSQC spectra of ^15^N-labeled integrin α1-TMC with different concentrations of non-labeled β1-TMC were acquired and the processed spectra are shown in Figure 4. Surprisingly, no obvious chemical shift perturbation was observed anywhere in the spectrum, indicating no pronounced conformational change of integrin α1-TMC upon addition of β1-TMC, maintaining the major helical conformation in the GFF motif. However, intensity attenuations were observed in several resonances with increasing integrin β1-TMC concentration (Fig. 4). These resonances with attenuated intensities were mapped to two regions: the N-terminal juxta-transmembrane region (V1140, W1143, V1144, I1145, S1148, A1151, and G1152) and the C-terminal juxta-transmembrane region (L1161, A1162, L1163, W1164, K1165, I1166, G1167, F1168, F1169, K1170, and R1171). These residues are marked with arrows at the top of Figure 4.

According to solution NMR relaxation theories, peak intensity attenuation or missing peaks are attributed to intermediate-time scale (microsecond to millisecond) conformational exchanges [39], [40], [41]. Thus, intensity attenuation or the ‘disappearance’ of residues are hypothesized to indicate interaction and these residues are located in the direct interface between integrin α1/β1.

This hypothesized α1/β1 interaction interface is possibly similar to interactions in integrin αIIb/β3 and consistent with previous mutagenesis and deletion studies of integrin α1 [42], [43], [44]. In the complex structure of αIIb/β3 TMC, αIIb residues W968, G972, G976, L979, L980, and R995 are involved in the dimer interface [19], [20]. Their corresponding residues in α1 are V1144, S1148, G1152, L1155, L1156, and R1171. Here, the HSQC peaks of four of them (V1144, S1148, G1152, R1171) were obviously attenuated in the NMR titration experiment with integrin β1-TMC, while perturbations of the other two residues (L1155, L1156) were not apparent because they were crowded by L1158 and I1160. Also, the αIIb/β3 residues involved in the dimer interface are largely conserved in α1/β1. Those observations implied that the dimer interface of α1/β1 TMC is similar to that of αIIb/β3 TMC. Further structural studies of α1/β1 TMC complex will illustrate detail interaction surfaces. Residue mutations and deletions in the two regions have been reported to interfere with associations between α and β subunits and between integrin and cytoplasmic binding partners, thus interfering with downstream signal transduction, leading to inhibition of cell spreading and stress fiber formation [42], [43], [44]. Thus, titration results of integrin α1 with the addition of integrin β1-TMC provide preliminary insights about the interaction interfaces between the two proteins, and provide a basis for further detailed studies of signal transduction in fibrosis, angiogenesis, or cancer cells.

## Supporting Information

Figure S1 **Sequence alignment of 18 integrin α-TMCs (A) and topology of integrin α1-TMC (B).**
(TIF)

Figure S2 **The predicted secondary structure results of each residue calculated using TALOS+.**
(TIF)

Figure S3 **The HSQC spectrum of β1-TMC.**
(TIF)
