# Supplementary figures and images for: Integrin α1 Has a Long Helix, Extending from the Transmembrane Region to the Cytoplasmic Tail in Detergent Micelles
Source: PLoS One. 2013 Apr 30;8(4):e62954. doi: 10.1371/journal.pone.0062954 (PMC3639902; doi:10.1371/journal.pone.0062954)

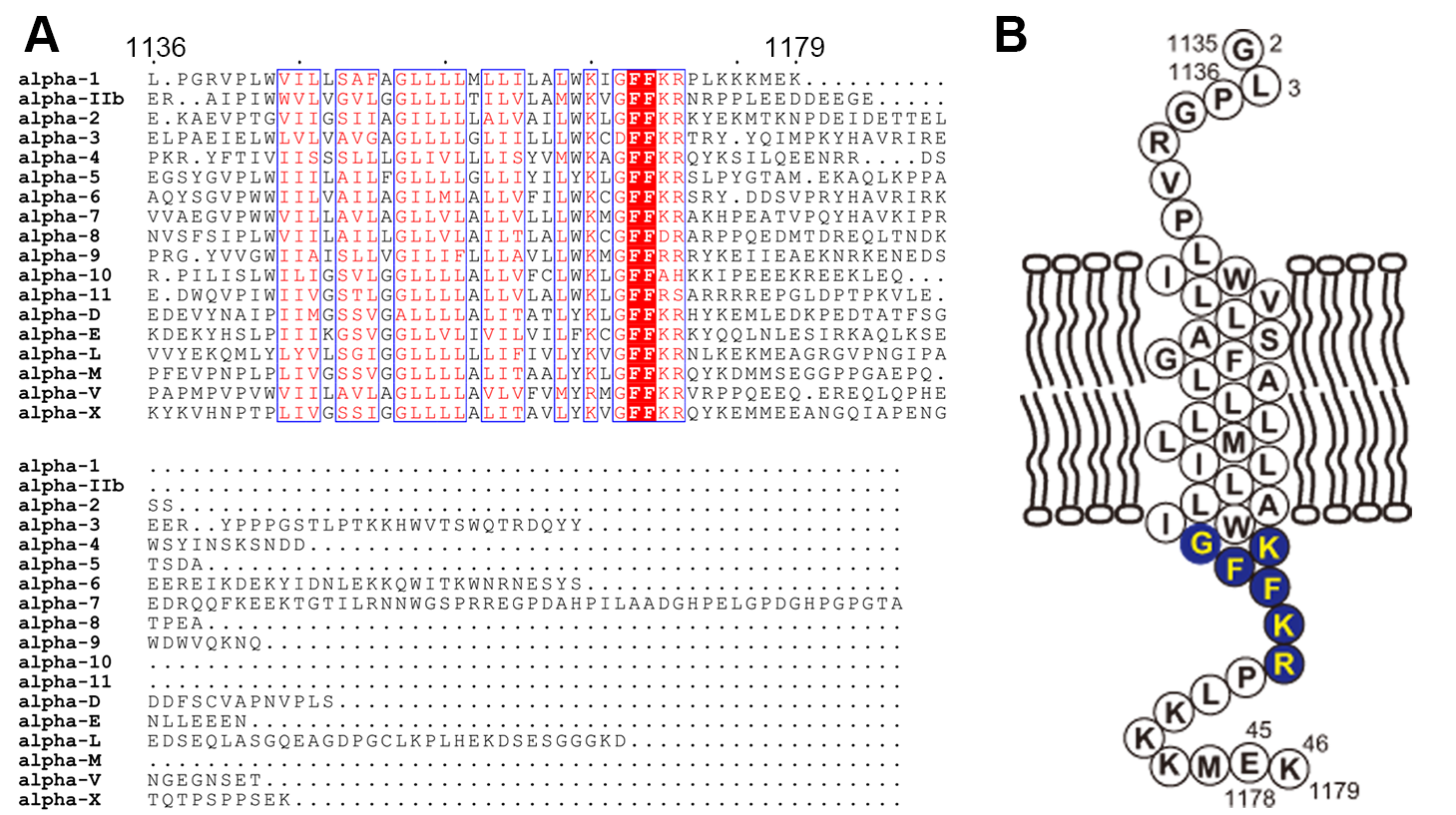

Supplement: Figure S1 — Sequence alignment of 18 integrin α-TMCs (A) and topology of integrin α1-TMC (B). (TIF) [file pone.0062954.s001.tif]

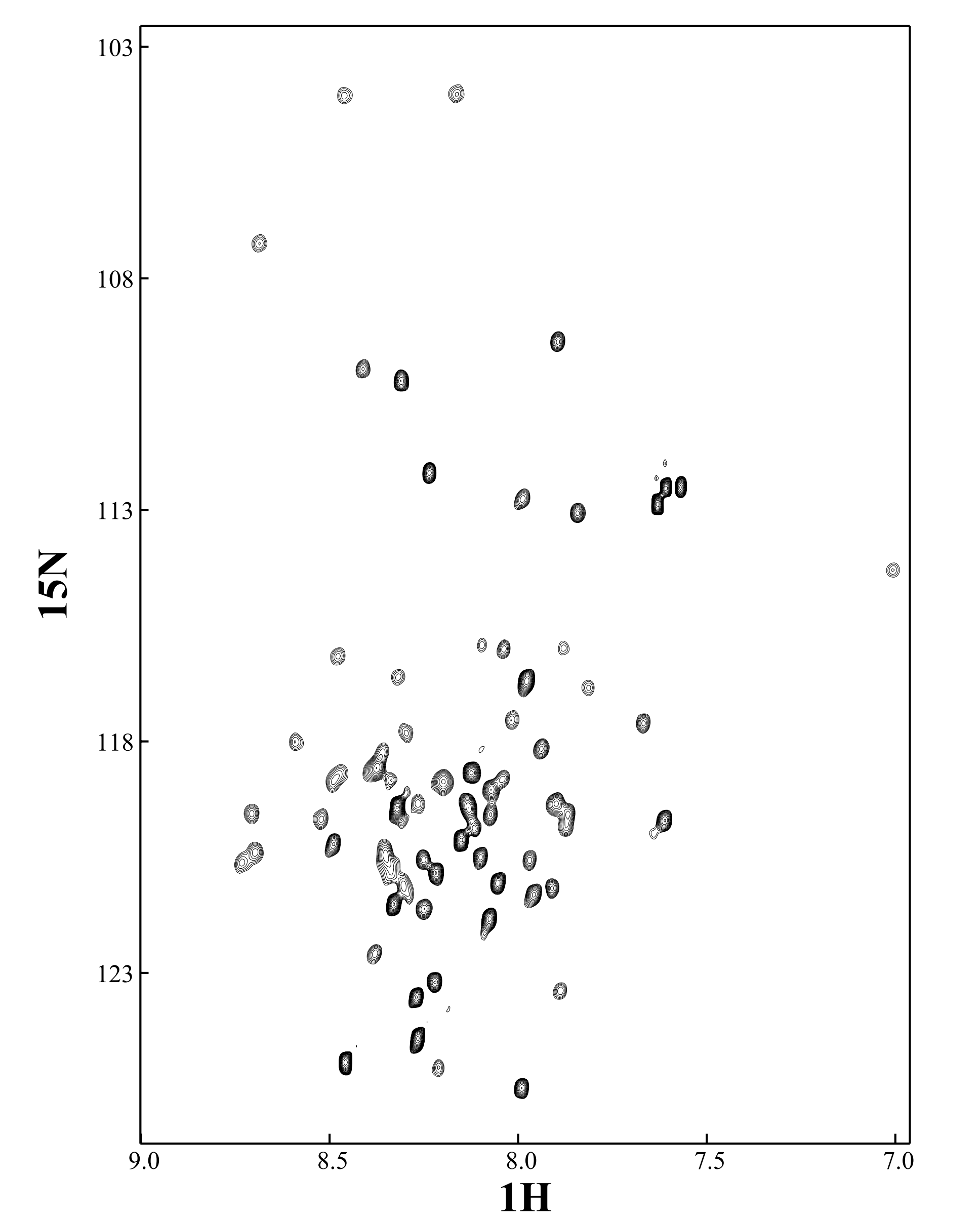

Supplement: Figure S3 — The HSQC spectrum of β1-TMC. (TIF) [file pone.0062954.s003.tif]
